# Practical challenges and Obligations for conducting Clinical Trial in Nepal: A call for improvement

**DOI:** 10.3126/nje.v9i3.25804

**Published:** 2019-09-30

**Authors:** Muhamed Ibnas, Mohammad Asim, Ahammed Mekkodathil, Brijesh Sathian

**Affiliations:** 1 Research Coordinator, Clinical Research, Trauma Surgery, Surgery Department, Hamad General Hospital, Doha, Qatar.; 2 Academic Research Associate, Clinical Research, Trauma and Vascular Surgery, Surgery Department, Hamad General Hospital, Doha, Qatar.; 3 Injury prevention coordinator, Clinical Research, Trauma and Vascular Surgery, Surgery Department, Hamad General Hospital, Doha, Qatar.

Clinical trials play a central role in human subject research for the betterment of health care outcomes. Clinical trials are based on experimental research design in which human subjects are being enrolled in a study arm to assess the safety and efficacy of an investigational product in a protocolized manner. Clinical trials are mainly designed to study novel therapeutic interventions in human subjects enrolled with predetermined criteria to look for the desired outcomes [1]. Depending upon the need, the clinical trial can utilize the randomized or non-randomized controlled methods which have their own implications in different settings.

Earlier most of the clinical trials are being conducted in the developed countries; however, this paradigm has changed over the years. During the past 10 years, clinical research industry is focusing towards the developing nations which offers larger heterogeneous population and is cost-effective as compared to western countries [2]. Therefore, outsourcing of clinical trials is attaining momentum in south Asian region, due to availability of treatment-naive patient pool, trained professionals with superior clinical infrastructure and lower cost [1,2]. In comparison to other South Asia countries, the health sector research in Nepal is slowly attaining pace in the recent years [3]. Therefore, skill development should be prioritized in Nepal to conduct high quality research trial by trained healthcare professional for ethical conduct of human subject research which will help in overcoming the various issues of research misconduct reported from the developing nations [4].

Clinical Trials for an investigational product are often sponsored by pharmaceutical company but are also initiated by academic institutions to know whether the available treatment or intervention works well or safe for a specific study population. Earlier audits of clinical trials have revealed several misconducts such as conducting research without ethics approval, subject enrolment without obtaining informed consent and data fabrication [5]. In order to overcome such misconducts, and improve transparency in clinical trial, prospective registration of clinical trial in an established registry is important obligation of research team.

Moreover, conduction of clinical trials necessitates clinical as well as research expertise for ethical conduct of research as per the ICH-GCP guidelines which sometimes remains challenging in clinical settings with limited resources and over-burdened clinical practice [6]. Notably, development of trial protocol, training study personnel, clinical trial initiation, monitoring and completion requires substantial amount of time and manpower. Therefore, a dedicated research team involving study investigators, research coordinators, research nurses, and clinical pharmacist should be well oriented and trained with study protocol related documents, procedures and regulatory guidelines for conducting research to avoid protocol deviations. The research support team should also remind the physicians to identify potential subjects to boost subject recruitment and completion of study documentation. It is mandatory to have delegated research team members for study related activities and follow-up visits as per protocol. There were situations where physicians are reluctant to participate in research leading to slower recruitment that build up enrolment pressure on study coordinator which may lead to protocol deviations/violations [7].

Particularly, in the developing countries there are certain challenges and difficulties attributed to the process of Institutional review board (IRB) approval [8]. Often study sites are struggling with delayed approvals or communications from regulatory authorities or IRBs which will affect the study initiation and conduction. Other, challenges are being observed during the conduct of clinical trials such as slow recruitment, no informed consent, incomplete information, improper documentation, lost to follow-up, and delayed study closeout which are likely to be attributed to the complex administrative proceedings [9].

Informed consent form is a mandatory document to be signed by the patient with voluntary participation after being fully informed about the study [10]. Patient should get ample time to read, understand and to come with any doubts for clear clarification, before participation [11]. Patient recruitment and retention are other challenges which sometimes lead to ethical misconduct for recruitment of ineligible subjects or inclusion of subject without obtaining consent. Also, lack of awareness of study protocol may lead to protocol deviation or violation. So, the study team should be well trained to follow the study protocol and institutional regulatory guidelines.

Particularly, in research in emergency settings require different consent forms such next of kin consent, deferred consent, and delayed patient consent [12]. Deferred consent or next of kin consent are being used in situations where the research is time critical subject has impaired cognition to decide for participation. In some cases, after obtaining the deferred or next of kin consent the subject may withdraw consent upon regaining consciousness. So, subject autonomy should be respected throughout the conduct of trial.

The Investigational Product/device are sometimes not maintained or stored properly that may affect the study quality. Moreover, clinical pharmacist is responsible for drug dispensing without prior training which may result in non-compliance. For international trials, investigational products are being dispensed with the help of electronic Interactive Voice Response System (IVRS) or an Interactive Web Response System (IWRS) to avoid misconduct [13]. Therefore, the study coordinator should be well trained to perform randomization and emergency unblinding with electronic system.

Data must be stored and secured in a logbook and password protected computer with the project manager at the research office. Hard copies must be stored in closed envelopes in locked cabinet. Highest accuracy and confidentiality of patient details should be maintained by the investigation team. Therefore, only lead PI and the designated team members should have access of the de-identified information. After study closeout, the study all documents should be stored as per regulatory requirements and the link between code and subject identifier should be destroyed. As per the international regulatory guidelines, the study documents should be archived for 5-25 years after study completion which requires dedicated space to maintain the privacy and integrity of the study data. Auditing and monitoring is being done by the research governing bodies to assess the progress of the clinical trial at regular intervals to ensure patient’s rights, safety and wellbeing, data safety, protocol compliance, study outcomes [14].

In conclusion, over the three decades, the healthcare research in Nepal has steady improved with the help of international collaborations. Also, there is a strong need to foster the culture of scientific research and capacity building through appropriate training to develop protocol, ethical conduct of trials and compliance with regulatory guidelines, and study protocols. Moreover, the healthcare research activities in Nepal should be performed under the strict guidance of Nepal Health Research Council (NHRC) in order to perform quality research [15]. So, there is a need to establish high quality research through creation of research database for health research and strengthening the national research institutions such as Nepal Injury Research center (NIRC) to improve evidence-based healthcare research.
